# Predicting One’s Turn With Both Body and Mind: Anticipatory Speech Postures During Dyadic Conversation

**DOI:** 10.3389/fpsyg.2021.684248

**Published:** 2021-07-13

**Authors:** Peter A. Krause, Alan H. Kawamoto

**Affiliations:** ^1^Department of Psychology, California State University Channel Islands, Camarillo, CA, United States; ^2^Department of Psychology, University of California, Santa Cruz, Santa Cruz, CA, United States

**Keywords:** articulation, motor control, speech planning, timing prediction, turn-taking

## Abstract

In natural conversation, turns are handed off quickly, with the mean downtime commonly ranging from 7 to 423 ms. To achieve this, speakers plan their upcoming speech as their partner’s turn unfolds, holding the audible utterance in abeyance until socially appropriate. The role played by prediction is debated, with some researchers claiming that speakers predict upcoming speech opportunities, and others claiming that speakers wait for detection of turn-final cues. The dynamics of articulatory triggering may speak to this debate. It is often assumed that the prepared utterance is held in a response buffer and then initiated all at once. This assumption is consistent with standard phonetic models in which articulatory actions must follow tightly prescribed patterns of coordination. This assumption has recently been challenged by single-word production experiments in which participants partly positioned their articulators to anticipate upcoming utterances, long before starting the acoustic response. The present study considered whether similar anticipatory postures arise when speakers in conversation await their next opportunity to speak. We analyzed a pre-existing audiovisual database of dyads engaging in unstructured conversation. Video motion tracking was used to determine speakers’ lip areas over time. When utterance-initial syllables began with labial consonants or included rounded vowels, speakers produced distinctly smaller lip areas (compared to other utterances), prior to audible speech. This effect was moderated by the number of words in the upcoming utterance; postures arose up to 3,000 ms before acoustic onset for short utterances of 1–3 words. We discuss the implications for models of conversation and phonetic control.

## Introduction

Successful spoken communication requires navigating two overlapping sets of temporal constraints. On the one hand, there is what might be called phonological timing: how the flow of articulatory events gives rise to intelligible speech. Without proper phonological timing, the intended utterance “dab” might be distorted to “bad” (Browman and Goldstein, 1995). On the other hand, there is what might be called situational timing: how phonetic events are timed against the background grid of the environment, including others’ speech. Situational timing is key to inter-speaker coordination. For example, inter-turn gaps at changes of floor are quite short, with mean gap time varying from 7 to 423 ms across several languages (Stivers et al., 2009). As we will outline below, most extant speech models assume that phonological and situational timing are governed by distinct cognitive mechanisms. We will argue that understanding inter-speaker coordination requires re-evaluating this assumption. Such coordination may arise when speakers apply situational timing mechanisms to aspects of the utterance traditionally viewed as the domain of phonological timing.

Traditional models assume that utterance initiation is controlled by an online decision mechanism sensitive to situational factors like a “go” signal (Sternberg et al., 1978), or, when adapted to the context of conversation, another speaker’s communicative cues (e.g., Levinson and Torreira, 2015; Levinson, 2016). However, once initiated, an utterance’s internal timing is assumed to follow a prefabricated motor plan. In Levelt et al.’s (1999) influential model, this plan is a programmatic gestural score produced by the phonetic encoding mechanism. In Articulatory Phonology with Task Dynamics (AP/TD), this plan comprises the parameterized constriction gestures (Saltzman and Munhall, 1989), the phase couplings between gestural planning oscillators (Saltzman and Byrd, 2000), and the π-gestures implementing prosodic adjustments at phrase boundaries (Byrd and Saltzman, 2003).

There is empirical support for the separability of speech planning and speech triggering, both in pure laboratory tasks and in conversation tasks. For example, delayed naming tasks (e.g., Sternberg et al., 1978) attempt to isolate the speech triggering process by informing the participant of what they will say ahead of time, and then providing a secondary “go” signal to cue speech onset. The assumption is that participants will withhold the articulatory response until the “go” signal. Contrariwise, speeded naming tasks (e.g., Meyer, 1990, 1991) attempt to isolate the planning process by asking participants to respond as quickly as possible after the content of the next utterance is revealed. The assumption is that participants will complete planning and then initiate articulation as quickly as possible afterward. In delayed naming, participants generally reserve acoustic onset until after the “go” signal, and produce shorter acoustic latencies compared to speeded naming tasks. Both phenomena fit with the claim that triggering has been isolated. Similarly, in conversational tasks, EEG evidence suggests that relevant speech planning begins long before a partner finishes their current utterance (Bögels et al., 2015). However, as mentioned above, inter-turn gaps are short; further, Levinson and Torreira (2015) indicate that acoustically overlapped speech comprises less than 5% of total conversation time. In aggregate, this suggests that speakers in conversation plan upcoming utterances and then acoustically withhold them while awaiting the next speech opportunity. Not only does the evidence converge to the conclusion that planning and triggering are separable, but it also implies possible parallels between delayed naming and conversational speech initiation. We will return to this point later.

The separability of speech planning and speech triggering does not, on its own, entail the strict encapsulation of phonological from situational timing. Instead, we propose that these ideas have been accidentally conflated, partly because of the classical “motor program” concept. Work in delayed naming has revealed evidence that speakers preferentially “chunk” their utterances during the final moments of preparation. Sternberg et al. (1978) found acoustic response latency following the “go” signal to be a linear function of the number of stress-bearing syllables in the utterance. This work was later replicated and extended by Wheeldon and Lahiri (1997). The finding has been offered as evidence that stress-bearing syllables are the “subprograms” of speech, terminology which certainly implies fixed movement timing. Arguably, however, this interpretation reflects a preexisting commitment to the computer metaphor, as much as it reflects the specific empirical evidence.

The notion of “soft” movement plans is already well-ensconced in the phonetics literature, in the form of AP/TD’s articulatory gestures. In that theory, planned gestures do not uniquely determine the spatial trajectories of articulators. Each gesture corresponds to an articulatory synergy (e.g., Browman and Goldstein, 1989); if the motion of one articulator is impeded, other articulators in the synergy can move differently to compensate. This affords the flexibility to adapt to unexpected situational events (such as perturbation of jaw motion during a bilabial closure, e.g., Folkins and Abbs, 1975; Kelso et al., 1984). Specific kinematic trajectories are emergent from the intersection of the gesture with the (dynamic, evolving) embedding context. It seems possible, at least in principle, that a plan for phonological timing could similarly comprise constraints (e.g., on the ordering and/or permissible overlap of actions) rather than a rigid specification of the behavioral time course. [Note that this is admittedly not the case in AP/TD itself; timing in that theory is prescribed by gestures’ stiffness parameters, combined with the stable phasing relationships of coupled planning oscillators. But other touchstones exist. See, for example, Jordan, 1986; Liu and Kawamoto, 2010; Tilsen, 2016, 2018. This narrative was recently reviewed in detail by Krause and Kawamoto (2020b)].

These issues are highly relevant to the triggering of speech in conversation. Conversational utterance timing is precise. This is true not only for canonical turns (as represented by extremely short inter-turn gaps), but also for backchannels, which tend to be acoustically initiated following similar syntactic and prosodic cues as floor transitions (e.g., Koiso et al., 1998; Ward and Tsukahara, 2000) and which have a proper timing that is both perceptible and non-random (Poppe et al., 2011). One possibility is that this precision is aided by mechanisms that predict opportunities for speech onset. Most of the relevant evidence comes from the turn-taking literature. De Ruiter et al. (2006) found that participants could predict the timing of turn ends from lexico-syntactic cues. Magyari and de Ruiter (2012) found that listeners partly predicted turn-end phrasings and suggested this prediction could be used as a proxy estimate of remaining turn length. Rühlemann and Gries (2020) gave evidence that speakers progressively slow speech rate over most of the turn, implying that listeners might use prosodic cues in turn-end prediction. However, contrary to the above, Bögels et al. (2015) reported that accurate turn-end detection required participants to hear turn-final intonational phrase boundaries.

Often overlooked is that the debated role of prediction in speech triggering is entangled with the issue of whether planning uniquely determines phonological timing. Before producing the earliest sounds of the utterance, speakers must first establish the initial constrictions in the vocal tract. Estimates and measurement practices vary, but Rastle et al. (2005) found the mean delay between articulatory and acoustic onset to range from 223 to 302 ms across syllable onset types. If speakers precisely time acoustic onset (e.g., by targeting no acoustic gap and no acoustic overlap at turn transitions, Sacks et al., 1974), they must work around this lead time. If this lead time is fixed by prior planning, a tricky problem arises. If a speaker waits to be certain a speech opportunity has arisen, they may initiate their utterance too late. The (un-compressible) lead time will then compound the delay preceding their acoustic response. If a speaker initiates their utterance from the predicted timing of a speech opportunity, then error in this prediction may lead them to start too early. The (un-expandable) lead time will then inexorably unfold to the point of an acoustic interruption. On this latter basis, Torreira et al. (2015) have argued that articulation is not initiated from predicted timing. Levinson and Torreira’s (2015) and Levinson (2016) model of turn taking asserts that utterances are largely planned on a partner’s turn but held in abeyance until the end of that turn is definitively detected.

To our knowledge, only one study has directly evaluated the late-initiation assumption using a conversational task. It therefore warrants specific consideration here. Torreira et al. (2015) analyzed breathing patterns of dyads during question-answer sequences. Specifically, they inspected the distribution of inbreath timings following the start of a question. This distribution was highly variable, but the mode fell 15 ms after question end. The authors reported this as evidence for late articulatory initiation. This study is an important first step in the area but has some critical limitations. One may question how well its restricted focus on question-answer sequences generalizes to both other kinds of turn exchanges and utterance types, such as backchannels, deliberately omitted from the turn-taking tradition. Further, we wonder whether the large variability in inbreath timings arose because a wider range of breathing strategies was in use than the study recognized. Finally, inbreath timing is not likely to index a fixed coordinative sequence for speech. For example, Mooshammer et al. (2019) found that acoustic response times were later for naming targets presented mid-inbreath, compared to ones presented mid-outbreath, suggesting speakers finished in-progress inhalations before initiating verbal responses. However, speakers also did not take new inbreaths, when presented with the target during exhalation.

Moreover, in naming research, articulatory kinematics often tell a different story from other measures. We earlier noted that conversational speech triggering invites comparison to the delayed naming paradigm. Classical findings in delayed naming, based on acoustic measures, appeared to indicate that speech was not initiated until the “go” signal. However, when delayed naming experiments have added articulatory measures, the results have suggested a different interpretation, one seemingly incompatible with the fixed-time-course narrative. Both Kawamoto et al. (2014) and Tilsen et al. (2016) presented participants with monosyllables to be read aloud upon “go” signal presentation, while measuring articulator positions using either video or structural MRI. The “go” signal followed stimulus presentation by a variable delay. Participants postured their vocal tracts to anticipate form-specific requirements of the utterance. They formed and maintained these postures during the unpredictable period separating stimulus onset from “go” signal, while nonetheless delaying the acoustic response until appropriate.

This suggests that speakers in conversation may have heretofore unrecognized degrees of freedom for coordinating acoustic onset timing. The silent interval during which initial constrictions are formed may in fact be compressible or expandable, even after movement has started. This leads us to the following general hypothesis motivating this study: We propose that speakers in conversation can initiate the earliest articulatory movements from predicted timing, at least under some conditions. We further suggest that they compensate for prediction error online, by slowing or speeding the articulatory time course as it unfolds. A second general hypothesis follows by implication from the first: The earlier that pre-acoustic articulation is initiated (with respect to the eventual acoustic onset), the lower the peak velocity of that motion.

This mechanism may not be equally utilized (or available) across all contexts. Laboratory work examining articulatory strategies for speech suggests they respond to many factors. Consider studies examining incrementality (i.e., speakers’ choices to produce speech by small chunks as they are planned, versus waiting and then producing large chunks all at once). Propensity to incremental speech can reflect individual differences (Kawamoto et al., 2014), subtleties of task (such as whether instructions were to begin speaking as soon as possible or to speak as briefly as possible, Holbrook et al., 2019), and even language spoken (Swets et al., 2021). Speaker’s use of predictive initiation with adjustment may therefore vary with several factors. These factors might include how early (with respect to the targeted moment of acoustic onset) the initial words of the utterance are planned, how much of the utterance can be held in working memory, and/or the speaker’s willingness to produce those early words incrementally. Overall, then, it may be that particularly short, stereotyped utterances are the most likely to be prepared in this manner, when disregarding other contextual factors. Knudsen et al. (2020) have recently suggested that speakers use such “forgotten little words” to mitigate conversational costs. The present study makes use of this observation.

We should emphasize that this tendency for articulatory preparation to arise more in some contexts than others is a core theme of the present study. It is not intended *per se* as a refutation of Levinson and Torreira’s (2015) proposed rule for articulatory initiation. It is more so intended as a fundamental reframing of the point, away from assertions of hard rules or typical cases, and toward consideration of the range of strategies available and their domains of application.

In the present study, we sought evidence for anticipatory speech postures in natural conversation. The study utilized data from the Cardiff Conversation Database (Aubrey et al., 2013), an audiovisual database of dyads engaging in unscripted conversations. Motion-tracked video of speakers’ faces was used to assess changes in lip area over time. We looked for contrasts between utterances beginning with smaller lip area due to closure and/or rounding (the *labially constrained* condition) versus other oral configurations (the *labially unconstrained* condition). Further, we examined how these contrasts were moderated by the number of words in the utterance. We specifically predicted that lip areas for labially constrained vs. unconstrained utterances would be discriminable earlier, relative to acoustic onset, when utterances were very short (1–2 words long).

## Materials and Methods

This study used data drawn from the Cardiff Conversation Database (Aubrey et al., 2013). This database is available by request from https://ccdb.cs.cf.ac.uk/signup.html. The authors asked several dyads to engage in 5-min unscripted conversations while their faces were video-recorded (at 30 frames per second) and their speech audio-recorded. While the authors suggested possible conversation topics, these topics were not actively enforced. Full data coding and transcription has been completed for eight dyads; these are the dyads analyzed in the current study.

### Participants

The eight analyzed dyads included six speakers. The following demographic information was provided by P. Rosin (personal communication, February 4, 2021; April 29, 2021). All speakers were Caucasian males. Ages ranged from 27 to 47 years (*M* = 36.33). Two speakers spoke English with a Welsh accent, one with a Scottish accent, one with a German accent, and two with an English accent (one having lived throughout Southern England, and one having grown up in Essex).

Each speaker participated in at least two dyads (equating to at least 10 min of recorded video footage). Details about the number of dyads each speaker participated in, plus additional information about their utterance distributions, appears in Table 1.

**TABLE 1:** Speaker-specific information.

Speaker	Number of dyads	Mins. of recordings	Constrained utterances	Unconstrained utterances
P1	2	10	12	29
P2	3	15	12	50
P3	3	15	50	59
P4	2	10	19	23
P5	2	10	14	36
P6	3	15	19	29

*Number of utterances refers only to retained data.*

### Elan Annotations

The database includes detailed, time-aligned behavioral annotations performed in the Elan software (Wittenburg et al., 2006) for all eight dyads. These annotations include temporal markings for the acoustic onsets and offsets of all verbal utterances, as well as full transcriptions of utterance content. Acoustic onsets for utterances were specifically marked at the first notable swell in audio intensity leading into an identifiable speech sound (P. Rosin, personal communication, April 29, 2021).

### OpenFace Tracking Outputs

The database authors processed each facial video using OpenFace 2.0 (Baltrušaitis et al., 2018). The database contains the resulting output files. OpenFace detects the most prominent face in a video and tracks its pose in six degrees of freedom relative to camera origin, as well as tracking the three-dimensional positions of 128 key points on the face. We have previously described how to extract linguistically useful information about oral configuration from OpenFace output (Krause et al., 2020). OpenFace’s positional estimates hew closest to the true values when OpenFace is provided with the camera’s intrinsic lens parameters. OpenFace was not calibrated in this way before processing the facial videos in the database (P. Rosin, personal communication, February 3, 2021). However, since all statistical inference will be based on within-speaker comparisons, the lack of a pure correspondence to real-world units is incidental.

**FIGURE 1:**
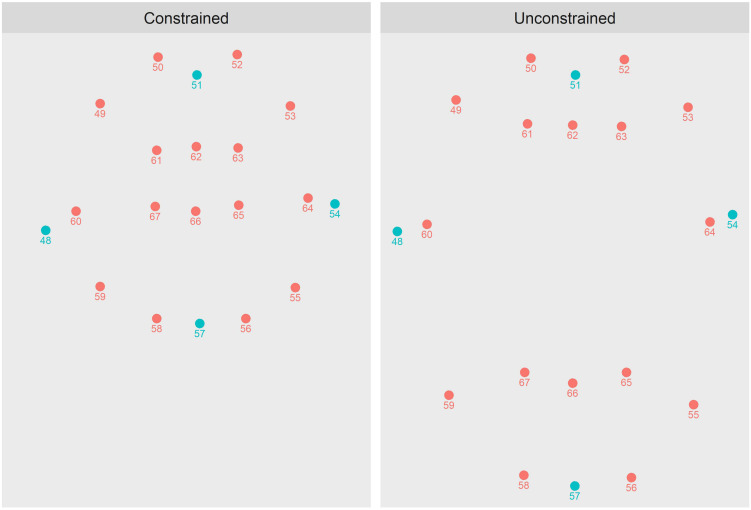
Plots of OpenFace parameters 48–67, which track the outer and inner lips, as arrayed on two frames of real facial video, each capturing the moment of acoustic onset for a different word. **(Left)** The first author beginning the word “oodles,” a constrained word. **(Right)** The first author beginning the word “apple,” an unconstrained word.

For readers unfamiliar with the OpenFace system, we provide Figure 1 as illustration. The panels of Figure 1 depict just those parameters of OpenFace that track the outer and inner lips (i.e., parameters 48–67). The dots colored in blue depict those parameters used in the lip-area computations described below. The panels specifically depict how OpenFace differentially tracks the lips when they are in different configurations (as in the *labially constrained* versus *labially unconstrained* utterance types, described in more detail below). To produce these plots, the first author spoke two example utterances on digital video that was later processed by OpenFace: the word “oodles” (a constrained utterance, left) and the word “apple” (an unconstrained utterance, right). The depicted tracking is from the moment of acoustic onset for both utterances. To facilitate comparison, both plots have been centered with respect to parameter 51, which marks the notch at the top of the outer lips.

### Data Preparation

In total, the Python script described below identified 509 distinct utterances in the annotated Elan data.

#### Utterance Types and Inter-Utterance Gaps

A specialized Python script processed the Elan output for each speaker. For each annotated utterance, the script determined the most recently initiated prior utterance (even if this prior utterance was not yet concluded). If that recently initiated utterance was by the same speaker, the present utterance was labeled as a restart and marked to be dropped from the final dataset (our interest being in inter-speaker coordination). Utterances were also marked as restarts if the most recent initiation was from the other speaker but had occurred within 500 ms of a prior initiation from the current speaker. Otherwise, utterances were marked as responses, and an inter-utterance gap time was computed by subtracting the onset time of the current utterance from the offset time of the most recently initiated prior utterance.

Similarly to Heldner and Edlund (2010), responses were further labeled as gaps (if the current utterance followed by previous one by a positive gap time), between-overlaps (if the current utterance started before the prior utterance completed, but finished afterward), and within-overlaps (if the current utterance lay completely within the acoustic boundaries of the prior utterance).

#### Lip Area Trajectories

By referencing the OpenFace outputs, the Python script computed lip area at each of 90 frames (3,000 ms) preceding the acoustic onset of each annotated turn. The script used the estimated *x*- and *y*-coordinates of four key points: The left-hand and right-hand corners of the lips (OpenFace parameters 48 and 54, respectively), and the external points at the center-top and center-bottom of the lips (OpenFace parameters 51 and 57, respectively). Area was computed as described by Liu et al. (accepted).

The strategy creates a perimeter of line segments running clockwise around the points, each conceived as the hypotenuse of a right triangle. Lip area is the sum of the areas of all four right triangles, plus the area of a residual central rectangle. Our formulas assume that one labels the left corner as (X_1_,Y_1_) and then increments the X- and Y-values while moving clockwise around the set. The following formula produces the areas of each right triangle *i* (which has one corner at (X*_*i*_*,Y*_*i*_*):

$$A_{i}=\frac{\left|X_{i}-X_{i+1}\right|\times \left|Y_{i}-Y_{i+1}\right|}{2}$$

The following formula produces the area of the residual central rectangle *j*:

$$A_{j}=\left|X_{1}-X_{3}\right|\times \left|Y_{2}-Y_{4}\right|$$

#### Identification of Key Predictor Values

The Python script also counted the number of words in the transcription for each turn and extracted the first transcribed word for reference.

We manually coded each turn as either *labially constrained* (in which case we would expect a comparatively small lip area at acoustic onset) or as *labially unconstrained.* We made this assessment based on the first syllable of the first annotated word. Specifically, we considered both the initial consonant (if applicable) and the nuclear vowel. Turns with initial consonants were classified as labially constrained if the consonant was bilabial, labiodental, or rounded, i.e., a member of the set /b, f, m, p, ɹ, v, w/. Regardless of initial consonant, turns were classified as labially constrained if their first nuclear vowel was rounded, i.e., a member of the set /ͻ, o, ʊ, u/. Turns not fitting either of these criteria were classified as labially unconstrained.

## Results

### Utterance Types and Inter-Utterance Gap Times

Restarts and the initial utterances of a conversation were expunged from the data, leaving 352 utterances in the set. Gap utterances comprised 47% of retained data, between-overlaps 22%, and within-overlaps 30%.

**FIGURE 2:**
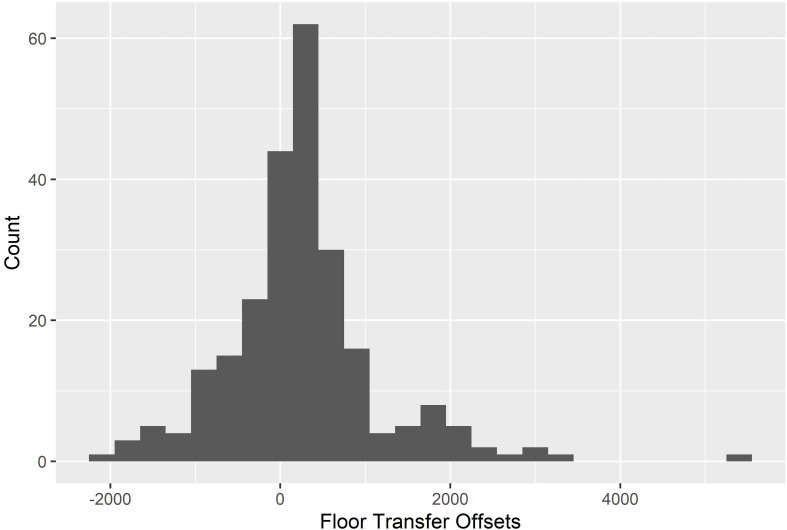
A histogram depicting the floor transfer offsets (i.e., the inter-utterance gaps for gap utterances and between-overlaps) in the final dataset.

Because the nominally intended start times for gap utterances and between-overlaps are relatively clear (i.e., the acoustic offset of the prior utterance) their coordination can be further characterized by considering their inter-utterance gap times (i.e., their floor transfer offsets, Levinson and Torreira, 2015). Figure 2 depicts a histogram of the floor transfer offsets in the final data. The mean floor transfer offset was 258.37 ms (SD = 914.96).

The coordination of within-overlaps cannot be so easily characterized, since these are typically backchannels or other short utterances whose timing targets are a syntactic or prosodic closure within an utterance that is acoustically continued. However, spot-checking of the transcript suggested that within-overlaps were coordinated reasonably.

For example, the most extreme within-overlap marked in the dataset arises in a conversation between P3 and P4. At this point, P4 has just been asked his favorite biography. Comparison of the Elan annotations with the acoustic waveforms reveals this rough pattern of coordination (bolded text indicates the specific point of overlap):

**P4**: The, the one on Copeland one Aaron Copeland one was a good one, **uhh**, a large volume…
**P3**: **Okay**.

P4’s turn continues for some time after without an acoustic break, resulting in the inter-utterance gap time for P3’s “Okay” being computed as −99 s, despite it having been realized shortly after the resolution of P4’s statement “Aaron Copeland was a good one.”

All within-overlaps were retained in the final dataset so as not to compromise statistical power for detecting anticipatory postures.

### Lip Area Trajectories

Statistical analysis of lip area was carried out in R version 4.1.0 (“Camp Pontanezen”), using the “lme4,” “lmerTest,” “interactions,” “effects,” and “bootMer” packages. We fit linear mixed-effects models to the data at 15-frame (500-ms) intervals, starting at 3,000 ms prior to acoustic onset, and ending at acoustic onset. This resulted in 7 fit models. The dependent variable in each case was lip area, with predictor variables of interest being utterance word count (log10-transformed to correct for extreme positive skewness), labial constraint (constrained vs. unconstrained), and their two-way interaction. We used Speaker ID and the first word of the utterance as clustering variables. We determined the random effects structure for each model via a backward selection procedure that started with the maximal structure (Barr et al., 2013) and then simplified until lme4 returned no warnings about convergence failure or model singularity. To facilitate hypothesis testing, we estimated denominator degrees of freedom via Satterthwaite’s method (see Luke, 2017 for a justification).

Our labial constraint predictor was dummy-coded “0” for constrained utterances and “1” for unconstrained utterances. Therefore, positive slope values would indicate larger lip area for unconstrained than constrained utterances, signaling the likely presence of speech postures. This gives the slopes of the word count × labial constraint interaction terms a straightforward interpretation. Negative values for these slopes would indicate decreasing probability and/or magnitude of speech postures with increasing word count, consistent with our hypothesis. When the interaction term was statistically reliable, we followed up by computing Johnson-Neyman intervals revealing the specific ranges of word count over which the labial constraint predictor differed from 0.

Table 2 reports the statistical results in detail, including the random effects structures of the final fitted models. Where we report the results of Johnson-Neyman intervals, we give ranges of actual word counts (as opposed to their log-10 transformations), and report only those ranges where the slope of labial constraint was both positive and within the observed span of the data.

**TABLE 2:** Reports of linear mixed models fit to lip area.

Time point (ms)	Model details	
−3,000	Random effects	(1 | speaker) + (1 | first word)
	Constraint × log10(word count)	β = −64.48, SE = 24.99, *t*(109.35) = −2.58*
	Main effect of constraint	β = 68.73, SE = 25.96, *t*(20.81) = 2.65*
	Johnson-Neyman interval for probable speech postures	[0, 3.09]
−2,500	Random effects	(log10(word count) | speaker)
	Constraint × log10(word count)	β = −61.86, SE = 24.14, *t*(291.77) = −2.56*
	Main effect of constraint	β = 66.33, SE = 23.62, *t*(251.79) = 2.81**
	Johnson-Neyman interval for probable speech postures	[0, 3.72]
−2,000	Random effects	(log10(word count) | speaker) + (1 | first word)
	Constraint × log10(word count)	β = −58.07, *SE* = 25.09, *t*(113.91) = −2.32*
	Main effect of constraint	β = 71.78, SE = 25.83, *t*(20.80) = 2.78*
	Johnson-Neyman interval for probable speech postures	[0, 4.47]
−1,500	Random effects	(log10(word count) | speaker)
	Constraint × log10(word count)	β = −90.33, *SE* = 25.56, *t*(299.03) = −3.53***
	Main effect of constraint	β = 105.23, SE = 24.96, *t*(253.49) = 4.22***
	Johnson-Neyman interval for probable speech postures	[0, 6.45]
−1,000	Random effects	(log10(word count) | speaker) + (1 | first word)
	Constraint × log10(word count)	β = −70.37, SE = 27.22, *t*(191.49) = −2.59*
	Main effect of constraint	β = 82.24, SE = 29.40, *t*(51.24) = 2.80**
	Johnson-Neyman interval for probable speech postures	[0, 3.89]
−500	Random effects	(log10(word count) | speaker) + (1 | first word)
	Constraint × log10(word count)	β = −42.84, *SE* = 28.79, *t*(277.68) = −1.49
	Main effect of constraint	β = 62.23, *SE* = 32.81, *t*(97.14) = 1.90^†^
	Johnson-Neyman interval for probable speech postures	N/A
0	Random effects	(log10(word count) | speaker) + (1 | first word)
	Constraint × log10(word count)	β = −35.16, SE = 24.35, *t*(267.60) = −1.44
	Main effect of constraint	β = 97.83, SE = 27.63, *t*(89.50) = 3.54***
	Johnson-Neyman interval for probable speech postures	N/A

*Random effects were first extracted as numeric vectors. Double-bar notation indicates uncorrelated random effects. Although Word Count was log10-transformed, Johnson-Neyman intervals are given here as number of words, for ease of interpretation.*

^†^*p* < 0.1.

*p < 0.05.

**p < 0.01.

***p < 0.001.

The pattern of results in Table 2 is consistent with our prediction that anticipatory postures would emerge earlier (resulting in earlier distinctions between labially constrained vs. unconstrained turns) for shorter utterances. The word count × labial constraint interaction was statistically reliable from −3,000 ms through −1,000 ms, with Johnson-Neyman intervals suggesting that postures were most likely for utterances of three words or fewer at the earliest time points. At the end of the time course this interaction (unsurprisingly) disappears, leaving only a reliable main effect of labial constraint at 0 ms.

**FIGURE 3:**
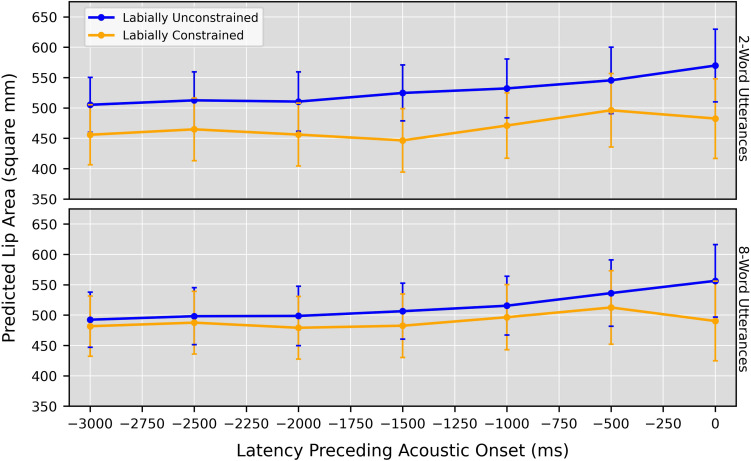
Predicted lip area values produced by the linear mixed models, when setting the word count predictor to 2 words and 8 words. Models were fit to junctures at 15-frame (500-ms) intervals, starting at 90 frames (3,000 ms) preceding acoustic onset. Error bars: Bootstrapped 95% CI.

Figure 3 presents a more visual illustration of these effects. For each of the seven models it plots the predicted lip area values (with bootstrapped 95% confidence intervals) for labially constrained and unconstrained utterances of two and eight words in length. (These values were chosen because two words is decidedly within the span at which the earliest anticipatory postures arose, while eight words is decidedly outside it, without being substantially larger).

### Maximum Lip Movement Speed

In the Introduction, we suggested that speakers might increase articulatory movement speed to partly offset delays from planning difficulty, meaning faster movement speeds would be expected for utterances of more words.

**FIGURE 4:**
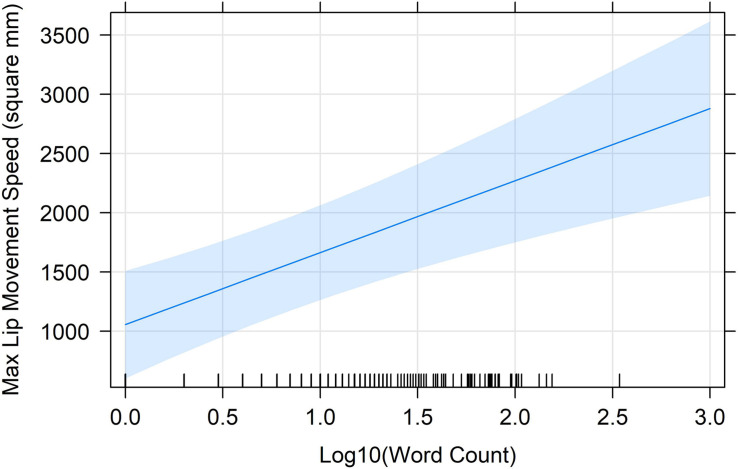
The regression line (with 95% confidence band) for the change of maximum lip movement speed with log10-transformed word count, as predicted by the mixed-effects model.

We analyzed lip movement speeds for all utterances. We did this by computing the change in area at each of the final 15 steps of the analysis window (when lips should be approaching final configuration targets) and multiplying each discrete change by 30 so that the units were given as mm^2^/s. We determined the maximum lip movement speed by selecting the resulting value with the largest absolute magnitude. We then fit a linear mixed-effects model as described above, with this absolute magnitude as the dependent variable. Log10-transformed word count, labial constraint, and their two-way interaction were the predictors. The final fitted model included random intercepts for Speaker ID and first word, and a random slope for labial constraint at the Speaker ID level. The only reliable effect was a main effect of word count (β = 455.22, SE = 185.15), *t*(64.73) = 2.50, *p* = 0.02. This effect suggests that, as predicted, lip movement speeds increase as word count increases. Figure 4 depicts the regression line for word count (with 95% confidence ribbon).

### Content of One-Word Utterances

The results suggest that the earliest anticipatory speech postures arose for utterances of three or fewer words. Although our hypothesis proceeded from the assumption that shorter utterances are easier to plan, there are multiple confounded reasons that this might be. For example, the smaller number of words might *per se* lower planning complexity, but the communicative content itself might also be simpler or higher in frequency. For this reason, some readers may wish to get a sense of the content of these shorter utterances. Table 3 presents a frequency-ordered list of every type of one-word utterance in the final dataset (one-word utterances representing 62% of all utterances of three or fewer words).

**TABLE 3:** Content and frequency of 1-word utterances.

Word	Count	Labial constraint
Yeah	37	Unconstrained
Alright	13	Constrained
Right	9	Constrained
No	8	Constrained
Yep	5	Unconstrained
Yes	4	Unconstrained
Really	3	Constrained
So	2	Constrained
Excellent	1	Unconstrained
Mmhmm	1	Constrained
Next	1	Unconstrained
Nice	1	Unconstrained
Oh	1	Constrained
Ok	1	Constrained
Thanks	1	Unconstrained
that’s	1	Unconstrained
Very	1	Constrained
Well	1	Constrained
What	1	Constrained
Which	1	Constrained

## Discussion

The main finding of the present study is that speakers awaiting their turn in natural conversation sometimes produce anticipatory oral postures well in advance of starting their acoustic utterance. In this study, statistical detection of these postures was facilitated when utterances were especially short (1–3 words). This may suggest that the articulatory planning and/or control of these postures is made easier when the overall utterance is low in complexity. As such, although this study extends the findings of a previous study of utterance timing in conversation (Torreira et al., 2015), it may not directly contradict that prior study’s finding that utterances were initiated late, since the utterances in the earlier study were of higher complexity.

Prior studies have reported similar effects for isolated word production tasks (Kawamoto et al., 2014; Drake and Corley, 2015; Tilsen et al., 2016; Krause and Kawamoto, 2019, 2020a; Tilsen, 2020). We believe, however, that this is the first report of such effects in an ecological conversation task. In addition, while Tilsen (2020) recently found that articulatory postures could arise as long as 500 ms before canonical articulatory onset, the current study is remarkable in finding that very short utterances could see postures arise as long as 3,000 ms before acoustic onset.

### Limitations

The non-experimental nature of our approach leaves the causal underpinnings of these postures underdetermined. We assumed that the short utterances would be faster to plan, leading to more cases of delay between phonological availability and acoustic response opportunity. However, the observed pattern may instead arise from some correlate of utterance length.

The analysis reflects 352 utterances produced by six male Caucasian speakers of English; generality may be limited. However, because anticipatory posturing has not previously been reported in an ecological task, the results are intrinsically important. They provide a valuable existence proof for the narrative of flexible articulatory control outlined in the Introduction, whereby speakers can independently manipulate utterance initiation and the time course of articulation. Further, the results suggest fertile possibilities for experimental follow-up.

### Theoretical Implications

We propose that, under some conditions, speakers can initiate articulation from ongoing prediction of the next speech opportunity, while using online control to finesse the moment of acoustic onset. Outstanding questions at this juncture include why postures are more likely under some conditions than others, and whether the postures are strategically functional.

#### Why Does the Emergence of Postures Vary?

As noted earlier, Torreira et al. (2015) concluded that articulation was initiated just after a partner’s utterance ended. Although this may reflect the specific dependent measure used (inbreaths, as indexed via inductive plethysmography), it may also be that the specific utterances analyzed did not facilitate early articulation. In the present study, anticipatory posturing only verifiably arose for utterances of 1–3 words in length. Although we predicted that postures should be variable, in accordance with the strategic flexibility observed in past articulatory studies, it remains uncertain exactly how this variability is structured.

We presumed that number of words in the utterance indexed planning complexity. If we are correct, then the phonology of more complex utterances might become available later, relative to the targeted moment of acoustic onset, leaving a shorter span inside which anticipatory postures could emerge and reduce movement speeds. Admittedly, however, “planning complexity” is ambiguous here. It could be that having fewer words to plan places less strain on the phonological system. It could also be that utterances that perform certain communicative functions or comprise certain high-frequency phrases are planned more easily, and that these utterances incidentally tend to be shorter. Further, the emergence of postures for certain kinds of utterances might reflect, not simpler planning, but some difference in either conventional timing or the need to visually signal intent (see below).

It might also be that the number of words in an utterance somehow moderates the readiness with which phonological plans are conferred into action. In their classic delayed-naming study, Sternberg et al. (1978) found that longer prepared utterances had longer acoustic latencies following the go signal. Although the present study is one of several to show that articulatory motion can be partly de-coupled from acoustic onset, perhaps this de-coupling becomes more difficult to manage as the upcoming utterance grows in length.

#### Are Postures Strategically Functional?

##### Possibility 1: intention leaks into articulation

One possibility is that the emergence of postures is not strategic but is instead a passive, incidental consequence of how the planning and motor systems are coupled. We base this possibility on Tilsen’s (2019) explanation of the anticipatory postures observed in laboratory tasks. In this account, the postures arise when partly activated but unselected speech gestures cascade their influences into current articulatory targets, across a partly permeable threshold.

This proposal can account for anticipatory speech postures in which the phonologically relevant constrictions are only partly formed (which Tilsen et al., 2016, found to be common). However, not all anticipatory postures are partial. In video recordings of their delayed naming task, Kawamoto et al. (2014) observed speakers who both closed their lips and accumulated intra-oral pressure when preparing /p/-initial utterances. (A comparable observation is not viable for the present study, owing to the small number of spontaneously produced utterances happening to start with bilabial plosives).

##### Possibility 2: reduction of movement costs

Starting early (i.e., lengthening posture duration) may minimize movement costs, as would be predicted by optimal control theories of speech behavior (e.g., Nelson et al., 1984). We observed that maximum lip movement speed was relatively lower over the ranges of word counts at which anticipatory postures arose. For a frictionless system, peak velocity is an estimate of the integral of force applied per unit mass with respect to time (Nelson, 1983). Such force integrals are one candidate approach for quantifying the energy and/or effort costs of motor function (see, e.g., Turk and Shattuck-Hufnagel, 2020).

##### Possibility 3: social signaling

Testing of Levinson and Torreira’s (2015) model has largely emphasized listeners’ abilities to predict and prepare for floor yielding. However, the anticipatory postures observed in this study may reflect the listener’s agency in effecting speech opportunities. It is possible that listeners deliberately use them to indicate readiness to speak. Irrespective of whether the behavior is deliberate, it may be perceived as a social signal by the current speaker. It might also assist the current speaker in managing attention, such that the incoming utterance is a less surprising event.

This proposal has some precedence. Kendon (1967) reported that as a speaker approached a possible floor transition, they tended to shift their gaze to the listener; this gaze continued for a while all the way through the transition. Listeners sometimes visually signaled a readiness to take the floor prior to the gaze shift. Similarly, Bavelas et al. (2002) found that brief windows of mutual gaze between speaker and listener often preceded backchannels. Kendrick and Holler (2017) presented evidence that listeners’ gaze patterns could influence the end of speakers’ turns. Averted gazes signaled that listeners were planning dispreferred responses; in some cases these gazes yielded last-minute repairs by the speaker intended to eliminate the dispreferred response.

### Concluding Remarks

The current study presented preliminary evidence that speech planning and articulation may flexibly overlap in natural conversation. This suggests that articulation may at times be initiated based on the predicted timing of speech opportunities, without obligating an acoustic interruption. Future work will be necessary to determine exactly what mechanisms speakers use to regulate the time course of articulation, how much deliberate strategy is involved, and whether this phenomenon carries social/pragmatic implications.

## Data Availability Statement

The datasets presented in this study can be found in online repositories. The names of the repository/repositories and accession number(s) can be found below: https://doi.org/10.17605/OSF.IO/JKR96.

## Author Contributions

PK carried out the data encoding and analysis and wrote the entire manuscript. Both authors jointly conceived and designed this study.

## Conflict of Interest

The authors declare that the research was conducted in the absence of any commercial or financial relationships that could be construed as a potential conflict of interest.
